# Supplementary material for: Privacy preserving data sharing method for social media platforms
Source: PLoS One. 2023 Jan 20;18(1):e0280182. doi: 10.1371/journal.pone.0280182 (PMC9858402; doi:10.1371/journal.pone.0280182)
Supplement: Attachment — Submitted filename: 18702.pdf [file pone.0280182.s001.pdf]

The authors have developed a new scheme for privacy preserving for data sharing to be used in healthcare applications. The following concerns must be addressed before final publication.

1. Introduction section must be strengthened exhibiting the scope and the motivations for developing the proposed system.
2. The objectives of the proposed system are not defined clearly.
3. Literature survey section must be extended. Some of the important manuscripts related to privacy preserving are not discussed. The titles of the related manuscripts are given below:
   - ✓ Computationally efficient privacy preserving anonymous mutual and batch authentication schemes for vehicular ad hoc networks.
   - ✓ Computationally efficient privacy preserving authentication and key distribution techniques for vehicular ad hoc networks.
   - ✓ EAAP: Efficient Anonymous Authentication With Conditional Privacy-Preserving Scheme for Vehicular Ad Hoc Networks.
   - ✓ CPAV: Computationally Efficient Privacy Preserving Anonymous Authentication Scheme for Vehicular Ad Hoc Networks.
   - ✓ Review: Privacy-Preservation in the Context of Natural Language Processing.
   - ✓ A secure and efficient authentication and data sharing scheme for Internet of Things based on blockchain.
   - ✓ Security and Privacy-Preserving Challenges of e-Health Solutions in Cloud Computing.
   - ✓ MGPV: A novel and efficient scheme for secure data sharing among mobile users in the public cloud.
4. The equations must be numbered that are used throughout the manuscript.
5. Table 2 is not visible completely.
6. Some graphical analysis during the comparative analysis must be shown for easy understanding.

Recommendation: Major Revision
